# Mission P(D)ossible: peritoneal dialysis in difficult cases

**DOI:** 10.1093/ckj/sfae403

**Published:** 2025-01-06

**Authors:** Kijanosh Lehmann, Benjamin Reubke, Reinhard Wanninger, Manuela Lindgren, Tim R Glowka, Jan T Kielstein, Gabriele Eden

**Affiliations:** Medical Clinic V, Nephrology, Rheumatology, Blood Purification, Academic Teaching Hospital Braunschweig, Germany; Department of General and Visceral Surgery, Academic Teaching Hospital Braunschweig, Germany; Medical Clinic V, Nephrology, Rheumatology, Blood Purification, Academic Teaching Hospital Braunschweig, Germany; Medical Clinic V, Nephrology, Rheumatology, Blood Purification, Academic Teaching Hospital Braunschweig, Germany; Department of General and Visceral Surgery, Academic Teaching Hospital Braunschweig, Germany; Medical Clinic V, Nephrology, Rheumatology, Blood Purification, Academic Teaching Hospital Braunschweig, Germany; Medical Clinic V, Nephrology, Rheumatology, Blood Purification, Academic Teaching Hospital Braunschweig, Germany

**Keywords:** catheter related infections, complications, kidney replacement therapy, patient autonomy, peritoneal dialysis

## Abstract

Indications for peritoneal dialysis (PD) have undergone a paradigm shift in recent years. Medical barriers previously viewed as contraindications for PD such as anuria, autosomal dominant polycystic kidney disease, cardiovascular diseases or advanced age are increasingly re-examined. We learned that establishing a safe, functional and durable catheter access can be established even in patients with a variety of co-morbidities. Moreover, conditions that predispose to catheter-related infections and peritonitis are not as obvious as we thought. In this case-based review we present patients who have been performing PD for a long time and in whom PD might still be viewed unfeasible. The aim of the paper is to underline the importance of PD as a kidney replacement therapy with fewer medical limits than previously thought. It is also a plaidoyer for interdisciplinary and interprofessional collaboration. This ‘Mission PD-possible’ should be accompanied by a coordinated approach aligning policy, organizational structures and financial resources.

## INTRODUCTION

Tom Cruise currently tries for the seventh time to do the impossible in the movie *Dead Reckoning.* According to Wikipedia, dead reckoning ‘is the process of calculating the current position of a moving object by using a previously determined position and incorporating estimates of speed, heading and elapsed time’.

In medicine we also know where we come from, and we are constantly moving, yet the direction is sometimes unclear. This also holds true for peritoneal dialysis (PD), an underutilized form of kidney replacement therapy (KRT). There are several context-specific barriers that need to be overcome. These include costs, healthcare resources, health system policies, provider bias and cultural beliefs [1]. The KDIGO controversies conference from 2023 highlighted that expanding home dialysis requires a coordinated approach, aligning policy, organizational structures and financial resources [1].

In addition to these non-medical barriers, there are medical conditions that may interfere with successful PD. These can be divided into contraindications to establishing a safe, functional and durable catheter access and conditions that predispose to catheter-related infections and peritonitis. Poor catheter choice can result in flow dysfunction, flow pain, and exit-site locations prone to infection or inconvenience to the patient. Furthermore, factors such as operator performance, obesity in the patient or previous peritonitis play a role in the outcome [2]. Moreover, being able to manually and intellectually perform the PD, such as lifting and operating the bags with PD fluid and connecting the tubing system is a prerequisite if assisted PD is not possible. A recent review on the topic identified the most important condition for PD aside from the patient’s preference—a functioning peritoneal membrane, which is a *conditio sine qua non* [3].

Whether PD is considered feasible or not also lies in the eye of the beholder. Directors of hemodialysis medical centers were less likely to recommend PD, showed less enthusiasm for PD and were more likely to cite patient preferences as a hindrance to PD growth [4].

**Figure 1:**
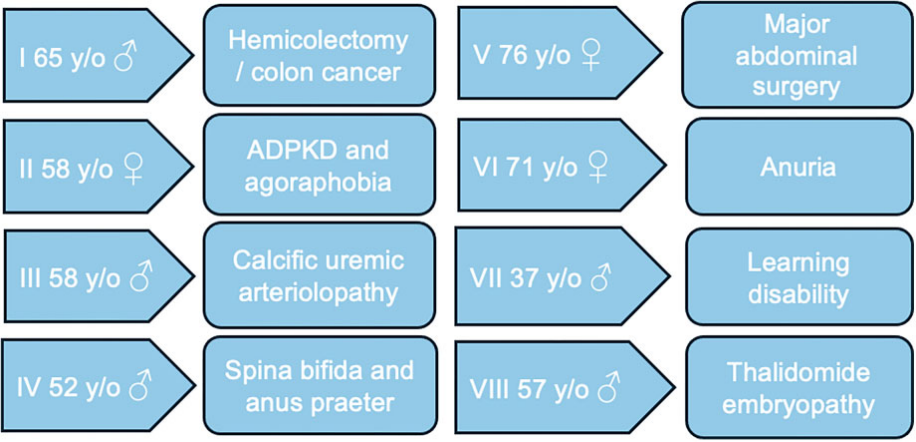
Conditions that make PD impossible (?).

This paper is not a comprehensive review but a collection of inspiring individual cases considering sparse data. It is also a plaidoyer for interdisciplinary and interprofessional collaboration. We would like to challenge the current general view on these conditions, turning a mission impossible into a mission PD possible (Fig. 1).

## PD possible Case 1: PD in a patient after hemicolectomy due to colon cancer

A 65-year-old female patient (BMI 27.0 kg/m^2^) with AKI was transferred by a primary care hospital, where she was hospitalized after myocardial infarction and percutaneous transluminal angioplasty. An ischemic and dilated cardiomyopathy was diagnosed (NYHA III, ejection fraction 30–35%, NT-proBNP 32 847 pg/mL), which contributed to a cardiorenal syndrome eventually requiring KRT. After informing the patient about all potential means of KRT, which is mandatory in Germany, the patient chose PD. A PD catheter was implanted by laparotomy.

With recovering residual diuresis and subsequentially improving renal function, the PD catheter could be removed 38 months after the initiation of PD. The patient did not require dialysis for almost 5 months, with the eGFR being 25 mL/min/1.73 m². Unfortunately, an adenocarcinoma of the ascending colon (TNM Classification pT3 (p) N0 0/17 M0 L0 V0) was diagnosed and removed by right hemicolectomy through a median laparotomy. In the postoperative phase kidney function deteriorated again, so dialysis had to be restarted. Due to the positive experience with PD the patient pursued this means of KRT. After a team discussion between surgery and nephrology a PD catheter was once again inserted. It required a revision due to leakage, a complication also seen in *de novo* PD catheter insertions in 5%–30% of cases [5, 6, 7]. This time a purse-string suture around the catheter exit from the intra-abdominal area around the PD catheter was applied. An extensive adhesiolysis was performed during the same surgical session. PD was restarted on the 5th postoperative day. The PD regimen was changed to CCPD and has since been carried out by the patient at home without any further complications. Despite hemicolectomy and several necessary surgical procedures on the abdomen, PD has continued to this day for more than 3 years.

In addition to the open surgical approach to colon cancer, there are cases in which a minimally invasive approach without removal of the PD catheter was used. Case reports from 2014 and 2016 suggest laparoscopic hemicolectomy to reduce the risk of need for HD transfer due to the formation of adhesions. Another advantage is the early resumption of PD [8], [9].

## PD possible Case 2: PD in a patient with autosomal dominant polycystic kidney disease (ADPKD) and agoraphobia

**Figure 2:**
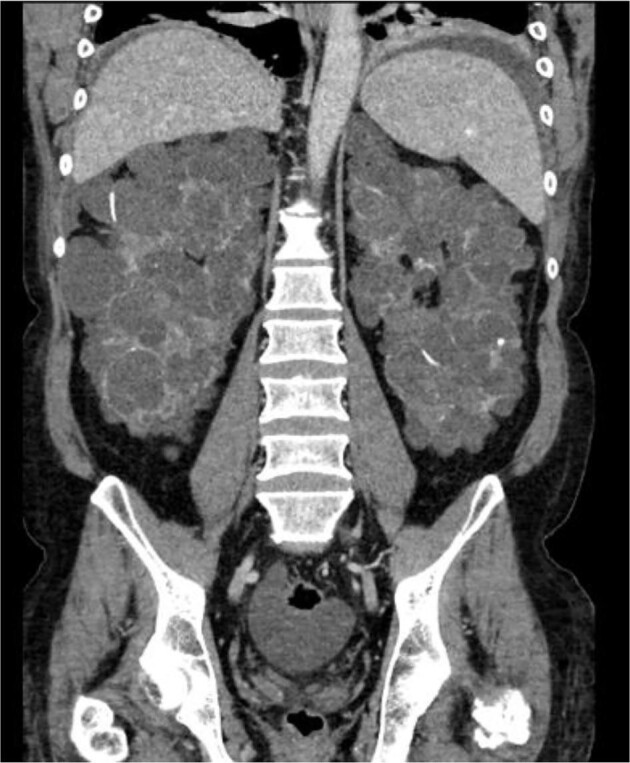
ADPKD and liver cysts.

A 58-year-old female patient (BMI 21.07 kg/m^2^) with ADPKD was referred to us. Due to ADPKD with associated liver cysts (Fig. 2) the outpatient nephrologist considered PD to be too difficult to implement and recommended hemodialysis. After 5 months of hemodialysis the patient developed symptoms such as tiredness, dyspnea on exertion, itching and restless legs syndrome. The intensity of the symptoms prompted the patient to pursue PD, as a dedicated PD nurse suggested that the symptoms might subside with PD, which was started after uncomplicated PD catheter insertion via laparotomy. CAPD could be performed up to a filling volume of 1000 mL without any complications. Appropriate filling volume was guided by regular measurement of the intraperitoneal pressure. The average intraperitoneal pressure we aim for is 10–16 cm H_2_O and should not exceed 18 cm H_2_O. If the intraperitoneal pressure is too high, complications such as hydrothorax or leakage could occur [10]. In our center, we put emphasis on intraperitoneal pressure measurement in ADPKD patients but recognize that it is currently neither widely adopted nor supported by robust data. It is surely no prerequisite to manage ADPKD patients on PD.

Due to recurring uremic symptoms, the regimen was changed to CCPD and has since been carried out by the patient at home without further complications. Fortunately, after initiation of PD, no symptoms emerged that could be attributable to increased intraperitoneal pressure, such as malnutrition or abdominal pain. A unilateral nephrectomy has so far been refused by the patient, but the dialysis effectiveness and ultrafiltration are more than adequate. Uremic symptoms have not occurred since. The patient does not suffer from edema, and hemoglobin levels and blood pressure are within the target ranges. Adding to the complexity is the fact that the patient suffers from anxiety disorder with agoraphobia. It is particularly important to her to perform dialysis independently at home. In addition, she has a phobia of needles, which would have made an HD transfer very difficult. The patients has now been on PD for 4.5 years.

Data from the French Language Peritoneal Dialysis Registry and the Renal Epidemiology and Information Network showed that only 10.9% of the ADPKD population are treated with PD [11]. The (regional) nephrologist's opinion about PD seems to play an important role in the underutilization of this KRT [12]. The view of surgeons has not been explored. A meta-analysis of 12 cohort studies including almost 15 000 patients on PD indicated that, compared with patients without ADPKD on PD, ADPKD was associated with significantly decreased mortality risk of 32%. Interestingly, ADPKD patients on PD were neither more prone to peritonitis nor to be switched to hemodialyis [13].

PD in ADPKD should be discussed on an individual basis, as is KRT in general. In contrast to a common misconception, recent data indicated that BMI and not cyst volume is the main factor linked to intraperitoneal pressure in patients with ADPKD [14]. A correlation between liver and kidney volumes and intraperitoneal pressure could not be found [15].

## PD possible Case 3: PD in a patient with calcific uremic arteriolopathy

A 58-year-old male patient (BMI 19.16 kg/m^2^) with biopsy-confirmed mesangioproliferative IgA glomerulonephritis chose PD as KRT.

Three and a half years into the PD treatment the patient presented to an external hospital with massively painful hip joints and hardening in that area. Calciphylaxis (calcific uremic arteriolopathy, CUA) was diagnosed by biopsy. The calcium phosphate product was increased (calcium 2.14 mmol/L, phosphate 1.92 mmol/L, PTH 85.9 pg/mL). To intensify dialysis a Shaldon catheter was inserted to temporarily add hemodialysis. Since the patient wished to continue PD, he was transferred to our center.

**Figure 3:**
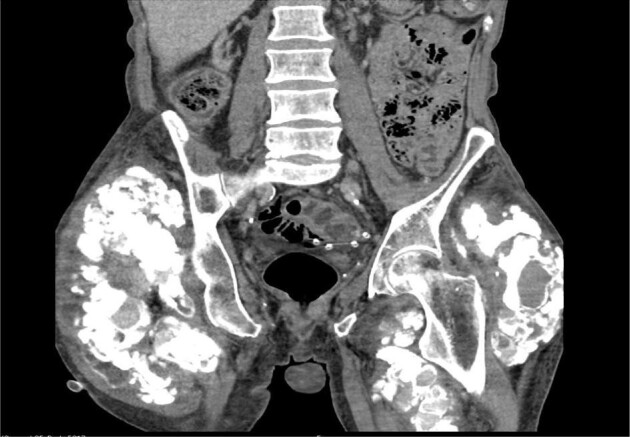
Massive calciphylaxis.

We intensified PD primarily through daily treatment with CCPD and regularly administered sodium thiosulfate (STS). STS was administered at a dosage of 20 g in 200 mL, yielding a 10% solution, for a total of 7 months and 2 weeks. The doses were given three times a week for 2 months and then two times a week for the rest of the time. A port system had now been implanted for parenteral administration of STS. The extraosseous calcifications (Fig. 3) had clearly regressed over the duration of treatment and the patient's pain had almost completely subsided. Hemodialysis was only necessary for 3 weeks.

STS for calciphylaxis treatment is more intensely studied in hemodialysis patients compared with PD patients. Nonetheless a systematic review showed 80% of patients achieving successful wound healing and 40% transition to hemodialysis [16]. While this review found that 20% of patients that received STS intraperitoneally died from chemical peritonitis [16], other authors consider this approach as a long-term strategy [17]. Although the use of intraperitoneal STS is viewed promising by a few authors, its application should be critically evaluated, as the risk–benefit ratio, particularly considering the potential for chemical peritonitis, is generally viewed as unfavorable.

The uncertainty surrounding the pathophysiology of calciphylaxis and the role of PD in its management is complex. In our case, the increase in clearance due to the higher frequency of PD may have played a role; however, clearance rates comparable to those achieved with daily HDF treatments were not attained. A clear agent triggering calciphylaxis, such as warfarin, was not present in our patient. Therefore, the added benefit of STS compared with increased clearance cannot be demonstrated. PD can still be a viable option when the triggers of calciphylaxis are not clearance-related or when the acute phase of the disease has subsided.

## PD possible Case 4: PD in a patient with spina bifida and anus praeter

**Figure 4:**
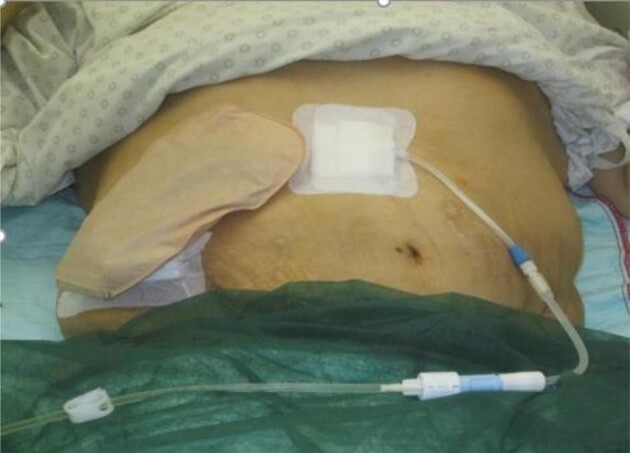
Coexistence of colostomy and PD catheter.

A 52-year-old multimorbid male patient (BMI 26.57 kg/m^2^) became dialysis-dependent due to chronic pyelonephritis secondary to a neurogenic bladder. He had received a total of three kidney transplants over a period of 7 years. The first two grafts became non-functional after just a few days due to acute rejection. The third graft was removed after 10 years due to chronic graft rejection and an abscess. Twenty-four years after initiating hemodialysis, through a native AV fistula, the patient had to be switched to PD due to the lack of vascular access options. PD was initiated as CCPD. The patient developed a progressive, deep sacral decubitus with osteitis of the ischial bone with non-closed spina bifida since birth. The defect had to be treated several times with wound debridement, vacuum-assisted closure therapy and a split-thickness skin graft. In the case of a fistula in the colon in the direction of the decubitus, a transversostomy had to be created. The PD could be continued despite a colostomy as CCPD, starting from the 6th postoperative day (Fig. 4).

There are sparse data on PD patients with a co-existing colostomy. From the International Pediatric Peritoneal Dialysis Network (IPPN) registry, 15 centers reported 20 children who received chronic PD with a co-existing colostomy [18]. In these children the annualized peritonitis rate was significantly higher in the colostomy group (1.13 vs 0.70 episodes per patient); however, age at the time of colostomy creation and PD catheter insertion were 0.1 and 2.8 months [18]. Data in adults are missing. In our view, if PD is done before formation of a colostomy patients should be offered appropriate counseling and continuation of PD, as in our case. Even in patients with pre-existing colostomy PD should not be ruled out in general.

## PD possible Case 5: PD in a patient after major abdominal surgery

A 76-year-old female patient (BMI 17.12 kg/m^2^) underwent PD for CKD5D as CAPD. In the 5th year of her PD treatment the patient was diagnosed with bilateral clear cell renal carcinoma. Both carcinomas were removed laparoscopically using extraperitoneal nephrectomy. PD could be continued. A year later PD was switched to hemodialysis as multiple complex polyps throughout the colon as well as multiple adenomas were planned to be removed by subtotal colectomy. During that procedure the surgeon noticed a recurrence of the renal carcinoma in the retroperitoneal space, which was removed in the same operation. After only 3 months, the dialysis procedure could be switched back to CAPD and continued without complications.

A recent review rightly summarized that a history of major abdominal surgery associated with extensive peritoneal membrane adhesions would be the main cause of losing the peritoneal membrane [3], yet even then PD might work, like in our case.

The case vividly demonstrates that PD may be feasible in certain cases despite repeated abdominal surgeries with associated adhesions. It also advocates interdisciplinary collaboration between nephrologists and general surgeons, emphasizing the crucial role of our surgical colleagues, who do not shy away from attempting PD catheter placement in patients with multiple previous abdominal surgeries.

## PD possible Case 6: PD in a patient with anuria

A 71-year-old female patient (BMI 24.54 kg/m^2^) required dialysis after allogeneic stem cell transplantation for myelodysplastic syndrome diagnosed 2 years earlier. In the aftermath of the stem cell transplantation, there was an anuric AKI stage 3 in the context of sepsis.

Initially dialysis was carried out as hemodialysis via a tunneled central venous catheter and then via a Cimino fistula, hoping that AKI would subside. After 1 year and 9 months of hemodialysis it was obvious that there would be no recovery, so, as part of a shared decision-making process, the dialysis procedure was switched to PD. Initially only low ultrafiltration rates could be achieved with PD.

In a peritoneal equilibration test, the patient turned out to have a fast peritoneal solute transfer rate. For this reason and because of the anuria, the PD mode was switched to CCPD. Adequate ultrafiltration rates could then be achieved with a shortened residence time of the peritoneal dialysate.

After several months of CCPD, the patient, who was anuric at first, experienced an increase in diuresis intensified by diuretic therapy. Today, the patient has a diuresis of 500 mL in 24 hours with a daily dose of 200 mg of torasemide. CCPD is performed with a 2.3% glucose solution and 7.7% icodextrin to enhance the osmotic effect, with the dialysate composition being adjusted to the clinical need. Additionally, the patient receives 25 mg of spironolactone daily to increase diuresis and to prevent hypokalemia, as well as negative chronotropic therapy with a β-blocker for atrial fibrillation. The case vividly demonstrates how patients on PD can experience an increase or even a recurrence of diuresis. The patient is currently still on CCPD.

## PD possible Case 7: PD in a patient with learning disability

We report on a male patient (BMI 30.49 kg/m^2^) with trisomy 21 who was 37 years old when dialysis was initiated. The patient was transferred to us with an infection, AKI and hypervolemia. Due to severe agitation and lack of cooperation, sedation was carried out to carry out hemodialysis via a Shaldon catheter after consultation with and consent of the legal guardian. The patient was diagnosed with CKD G 5 with severe glomerular global sclerosis on kidney biopsy most likely due to poorly treated arterial hypertension for many years.

After repeated in-depth discussion with the patient and his sister PD was pursued. To test whether the PD catheter would be tolerated an unsterile PD catheter was taped to the ventral abdominal wall *loco typico*. As this dummy was tolerated a PD catheter was inserted, which was also well tolerated. Despite increased care requirements, the patient was able to carry out PD for over 2 years before he died of severe sepsis with destructive abscesses in his shoulder and hip joints.

In cases of learning disability, hemodialysis is often given priority over PD. However, there are cases in which PD can be carried out successfully. A case report showed that PD could be carried out for 40 months in Prader–Willi syndrome with associated learning disability. Appropriate family support is essential [19].

An encouraging study in the pediatric field showed that rates of peritonitis and catheter-related infections were not higher in children with disabilities than in children without disabilities on PD [20].

## PD possible Case 8: PD in a patient with thalidomide embryopathy

We report on a 57-year-old male patient (BMI 31.45 kg/m^2^) with thalidomide embryopathy. The scientist was facing the start of KRT due to a congenital solitary kidney in the context of a thalidomide embryopathy, which also precluded a renal biopsy.

**Figure 5:**
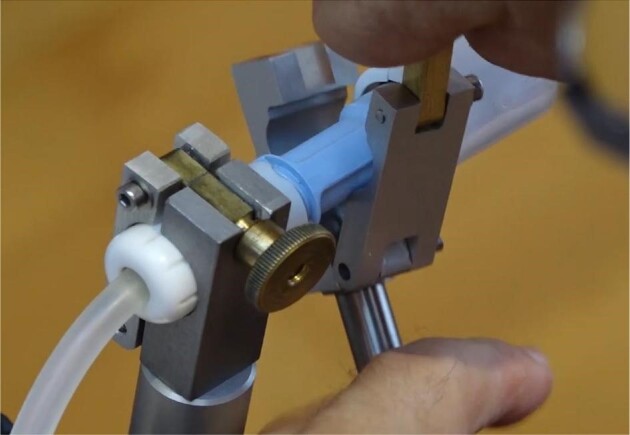
Device for connecting and disconnecting the PD bags.

Hemodialysis was out of the question for the patient not only due to the congenital malformations associated with thalidomide embryopathy, but even more so because autonomy was a priority for him, which he had pursued throughout his life against all odds. As part of a shared decision-making process we decided to initiate PD and implanted a PD catheter. Changing the bags for PD was a hurdle for the patient because of the amelia. To solve the problem, the PhD in physics developed a device for changing the bags of PD in cooperation with his employer, the national meteorology institute of Germany (Fig. 5). Nevertheless, due to his physical limitations, the patient relied on his wife's assistance for transporting and moving the PD bags.

The procedure was changed from CAPD to CCPD after a short time to minimize the frequency of bag changes. PD was performed without any problems. The supplementary video file gives an impression of the procedure.

The drug thalidomide, sold under the brand names Contergan^®^ and Thalomide^®^, was first marketed in 1957 in West Germany, where it was available as an over-the-counter drug to treat anxiety, sleeplessness and morning sickness. The total number of infants affected by thalidomide use during pregnancy is estimated at 10 000; most of them died around the time of birth. Almost 3000 patients did, however, survive with serious limb, eye, urinary tract and heart problems.

Rudimentary limb buds do not allow the formation of a regular vascular access for hemodialysis and PD is considered impractical because of the lack of arms [21]. With support of a skilled engineering and technical team PD was feasible in our patient.

In this context, expanding assisted PD, as practiced in some countries, would be desirable to provide patients without extensive knowledge and resources with the opportunity to live independently.

It will not be before 2025 that Tom Cruise will accomplish a mission impossible for the eighth time. Meanwhile, interprofessional and interdisciplinary teams around the world will do the impossible many times by enabling PD in those patients who have chosen this form of kidney replacement therapy. Maybe dead reckoning is outdated as a way of determining where we are in PD. Let us rather use GPS (**G**enius–**P**D **S**olutions).

## Supplementary Material

sfae403_Supplemental_File

## References

[1] Perl J, Brown EA, Chan CT et al. Home dialysis: conclusions from a Kidney Disease: Improving Global Outcomes (KDIGO) Controversies Conference. Kidney Int 2023;103:842–58. 10.1016/j.kint.2023.01.006 36731611

[2] Crabtree JH, Shrestha BM, Chow KM et al. Creating and maintaining optimal peritoneal dialysis access in the adult patient: 2019 update. Perit Dial Int 2019;39:414–36. 10.3747/pdi.2018.00232 31028108

[3] Lambie M, Davies S. An update on absolute and relative indications for dialysis treatment modalities. Clin Kidney J 2023;16:i39–47. 10.1093/ckj/sfad062 37711635 PMC10497377

[4] Shen JI, Schreiber MJ, Zhao J et al. Attitudes toward peritoneal dialysis among peritoneal dialysis and hemodialysis medical directors: are we preaching to the right choir? Clin J Am Soc Nephrol 2019;14:1067–70. 10.2215/CJN.01320119 31278114 PMC6625627

[5] Hu J, Liu Z, Liu J et al. Reducing the occurrence rate of catheter dysfunction in peritoneal dialysis: a single-center experience about CQI. Ren Fail 2018;40:628–33. 10.1080/0886022X.2018.1515084 30396302 PMC6225513

[6] Garcia Falcon T, Rodriguez-Carmona A, Perez Fontan M et al. Complications of permanent catheter implantation for peritoneal dialysis: incidence and risk factors. Adv Perit Dial 1994;10:206–9. 7999829

[7] Ouyang CJ, Huang FX, Yang QQ et al. Comparing the incidence of catheter-related complications with straight and coiled Tenckhoff catheters in peritoneal dialysis patients—a single-center prospective randomized trial. Perit Dial Int 2015;35:443–9. 10.3747/pdi.2013.00016 24584608 PMC4520727

[8] Auricchio S, Mari G, Galassi A et al. laparoscopic left hemicolectomy for colon cancer in peritoneal dialysis patients: a valid and safe surgical technique to ensure peritoneal dialysis survival. Perit Dial Int 2016;36:695–9. 10.3747/pdi.2015.00259 27903856 PMC5174882

[9] Attard JA, Attard A. Laparoscopic right hemicolectomy in an automated peritoneal dialysis patient without removal of the PD catheter: a case report. Case Rep Surg 2014;2014:492567. 25110601 10.1155/2014/492567 PMC4119649

[10] Perez Diaz V, Sanz Ballesteros S, Hernandez Garcia E et al. Intraperitoneal pressure in peritoneal dialysis. Nefrología 2017;37:579–86. 10.1016/j.nefro.2017.05.014 28739249

[11] Sigogne M, Kanagaratnam L, Dupont V et al. Outcome of autosomal dominant polycystic kidney disease patients on peritoneal dialysis: a national retrospective study based on two French registries (the French Language Peritoneal Dialysis Registry and the French Renal Epidemiology and Information Network). Nephrol Dial Transplant 2018;33:2020–6. 29361078 10.1093/ndt/gfx364

[12] Bouvier N, Durand PY, Testa A et al. Regional discrepancies in peritoneal dialysis utilization in France: the role of the nephrologist's opinion about peritoneal dialysis. Nephrol Dial Transplant 2009;24:1293–7. 10.1093/ndt/gfn648 19033252

[13] Boonpheng B, Thongprayoon C, Wijarnpreecha K et al. Outcomes of patients with autosomal-dominant polycystic kidney disease on peritoneal dialysis: a meta-analysis. Nephrology 2019;24:638–46. 10.1111/nep.13431 29952039

[14] Ferreira AC . Intraperitoneal pressure in peritoneal dialysis patients: a need for treatment individualization. Clin Kidney J 2023;16:1367–8. 10.1093/ckj/sfad140 37664561 PMC10469088

[15] Sigogne M, Kanagaratnam L, Mora C et al. Identification of the factors associated with intraperitoneal pressure in ADPKD patients treated with peritoneal dialysis. Kidney Int Rep 2020;5:1007–13. 10.1016/j.ekir.2020.04.012 32647758 PMC7335974

[16] Gossett C, Suppadungsuk S, Krisanapan P et al. Sodium thiosulfate for calciphylaxis treatment in patients on peritoneal dialysis: a systematic review. Medicina (Kaunas) 2023;59:1306. 37512116 10.3390/medicina59071306 PMC10386543

[17] Teh YK, Renaud CJ. Clinical experience with intraperitoneal sodium thiosulphate for calciphylaxis in peritoneal dialysis: a case series. Perit Dial Int 2023;44:66–9. 10.1177/08968608231163669. 37131321

[18] Chan EYH, Borzych-Duzalka D, Alparslan C et al. Colostomy in children on chronic peritoneal dialysis. Pediatr Nephrol 2020;35:119–26. 10.1007/s00467-019-04372-x 31673828

[19] Anno E, Hori K, Hoshimoto A et al. Successful peritoneal dialysis for the end-stage kidney disease associated with Prader-Willi syndrome: a case report. CEN Case Rep 2019;8:216–20. 10.1007/s13730-019-00395-3 30963414 PMC6620230

[20] Aksu N, Yavascan O, Anil M et al. Chronic peritoneal dialysis in children with special needs or social disadvantage or both: contraindications are not always contraindications. Perit Dial Int 2012;32:424–30. 10.3747/pdi.2009.00202 22045099 PMC3524858

[21] Gibbs PJ, Friend PJ, Darby CR. Renal replacement therapy in a patient with phocomelia. J R Soc Med 2003;96:558. 14594970 10.1258/jrsm.96.11.558 PMC539635

